# Genetic variants in human *BCL2L11* (*BIM*) are associated with ulcerative forms of Buruli ulcer

**DOI:** 10.1080/22221751.2021.1878936

**Published:** 2021-02-11

**Authors:** João Fevereiro, Alexandra G. Fraga, Carlos Capela, Ghislain E. Sopoh, Ange Dossou, Gilbert Adjimon Ayelo, Maria João Peixoto, Cristina Cunha, Agostinho Carvalho, Fernando Rodrigues, Jorge Pedrosa

**Affiliations:** a Life and Health Sciences Research Institute (ICVS), School of Medicine, University of Minho Braga, Portugal; b ICVS/3B's - PT Government Associate Laboratory, Braga/Guimarães, Portugal; c Centre de Dépistage et de Traitement de l'Ulcère de Buruli d'Allada, Ministry of Health, Allada, Bénin; d Institut Régional de Santé Publique, University of Abomey-Calavi, Ouidah, Bénin

**Keywords:** *Mycobacterium ulcerans*, Candidate gene, Single Nucleotide Polymorphism, rs13421194, BIM

## Abstract

Buruli ulcer (BU) is a devastating skin mycobacterial infection characterized by extensive cell death, which was previously suggested to be mediated by Bcl2-like protein 11 (BIM, encoded by the *BCL2L11* gene). We here report the association of genetic variants in *BCL2L11* with ulcerative forms of the disease in a cohort of 618 Beninese individuals. Our results show that regulation of apoptosis in humans contributes to BU lesions associated with worse prognosis, prompting for further investigation on the implementation of novel methods for earlier identification of at-risk patients.

Buruli ulcer (BU) is a skin disease typically prevalent in tropical countries, with over 20000 cases diagnosed in the past decade worldwide [1]. Although its global incidence has been decreasing, local foci of the disease in countries such as Ghana or Australia have been displaying an increasing number of BU notifications, leading to public health concern [1]. The disease is caused by *Mycobacterium ulcerans*, which produces mycolactone, a macrolide-like toxin that is able to induce cellular apoptosis [2]. Upon infection, patients develop a small skin lesion – nodule, ulcer, plaque or oedema – that slowly and painlessly may progress to larger disfiguring ulcerative lesions as a consequence of extensive cellular apoptosis and necrosis [2,3]. The transition to these more severe forms of the disease was shown to depend on variables other than time, likely related to the host–pathogen genetic interplay [3]. In this regard, mice with absent expression of pro-apoptotic mediator *Bcl2l11* revealed enhanced resistance to *M. ulcerans*-induced tissue ulceration [2]. Thus, we sought to determine whether genetic variants in human *BCL2L11* could associate with the risk of developing BU or the progression to ulcerative forms of the disease in a Beninese cohort.

We performed this study in accordance with the ethical standards of the Helsinki Declaration. The National Ethical Review Board of the Ministry of Health in Benin approved the collection and analysis of blood samples [clearance Nu 018, 20/Oct/2011; registration number IRB0006860]. We selected SNPs (Supplemental Table 1) based on their ability to tag surrounding variants in the HapMap-YRI population of the International HapMap project, phase III, NCBI (National Center for Biotechnology Information) build 36 [4]. To this aim, we used Haploview version 4.2 software [5] to assess linkage disequilibrium blocks with a pairwise correlation coefficient r^2^ ≥ 0.8 and a minor allele frequency (MAF) > 0.05, using the confidence intervals method and the LD-plot function. Considering a power estimation of 80% [6] – significance level of 0.01, additive disease model, disease prevalence of 0.02, MAF of 0.07, and genotype relative risk of 2.0 –, we expanded the cohort previously described in [7] with new cases diagnosed up until 2017, to a total of 618 age- and gender-matched cases (309) and controls (309). A final minimum call rate > 97% was obtained for all the SNPs genotyped. Using PLINK version 1.07, we assessed Hardy-Weinberg equilibrium with the χ^2^ test and performed genetic associations by means of logistic regression, under an additive model of inheritance and taking as reference the major allele [8]. Empirical *p*-values were further calculated through 5000 Monte Carlo permutations, provided the random seed “1473879600”, and adjusted for multiple comparisons. We explored SNP features using the Ensembl browser, release 100 [9]. Transcription factor binding sites (TFBS) were predicted with the PROMO software, using version 8.3 of TRANSFAC, and considering a maximum dissimilarity rate of 15 [10].

One SNP, rs1980045, displayed a significant deviation from the Hardy-Weinberg principle and was subsequently excluded from further analyses (*p* = <0.0001). Upon comparison of the genotype distribution across groups, according to the presence of BU history and the type of lesion manifested, we found the T allele at rs13421194 (OR = 2.017; 95% CI = 1.157-3.518; *p* = 0.013) to be associated with a two-fold increase in the odds of developing ulcers (Table 1). Intronic SNPs are increasingly being recognized as important modulators of genetic expression [11]. In silico analysis predicted rs13421194 to possess binding sites to Signal transducer and activator of transcription 4 (STAT4) and to c-Ets-1 (Supplemental Figure 1), which have been implicated in several cellular processes, including autophagy and mycobacterial growth inhibition [12], survival of lymphoid cells, regulation of cytokine and chemokine-related pathways, and apoptosis during the angiogenesis process [13]. We have also found that rs13421194 overlaps *MIR4435-2-HG* (MIR4435-2 host gene, ENSG00000172965.17) (Supplemental Figure 2), a long non-coding RNA (lncRNA) whose homologous in mice, *Morrid*, regulates the transcription of *Bcl2l11,* particularly in short-lived myeloid cells, both in physiological and infectious contexts [14]. On the other hand, lncRNAs have been found to strongly associate with the development of BU in a recent genome-wide association study [15]. These findings thus highlight novel putative mechanisms of regulation of *BCL2L11* expression during *M. ulcerans* infection, commendable of exploration in future studies.

**Table 1.** Genotype distributions and association test results of SNPs in the BCL2L11 gene among BU patients and healthy endemic controls (left) and among BU patients with non-ulcerative and ulcerative forms of the disease (right).

SNP rs#	Genotype	Cases (%)	Controls (%)	OR [95% CI]	*t*-statistic	*p*-value	Empirical *p-*value	Adjusted empirical *p-*value	Non-ulcerated (%)	Ulcerated (%)	OR [95% CI]	*t*-statistic	*p*-value	Empirical *p*-value	Adjusted empirical *p*-value
**rs1821968**	GG	133 (42.9)	131 (42.4)	0.913 [0.719-1.162]	-0.735	0.462	0.502	0.917	29 (36.7)	95 (44.6)	1.264 [0.851-1.878]	1.159	0.246	0.264	0.656
	GA	145 (46.9)	146 (47.7)						41 (51.9)	98 (46.0)					
	AA	32 (10.4)	32 (10.5)						9 (11.4)	20 (9.4)					
**rs17041869**	AA	260 (84.1)	257 (83.2)	1.040 [0.707-1.529]	0.197	0.844	0.795	0.999	63 (79.7)	183 (85.9)	1.461 [0.796-2.684]	1.223	0.221	0.206	0.596
	AG	45 (14.6)	49 (16.0)						15 (19.0)	28 (13.1)					
	GG	4 (1.3)	3 (1.0)						1 (1.3)	2 (0.9)					
**rs9308731**	AA	233 (75.4)	235 (76.1)	0.898 [0.650-1.239]	-0.656	0.512	0.508	0.925	64 (81.0)	156 (73.2)	0.697 [0.405-1.199]	-1.305	0.192	0.182	0.530
	AG	66 (21.4)	70 (22.9)						13 (16.5)	49 (23.0)					
	GG	10 (3.2)	4 (1.3)						2 (2.5)	8 (3.8)					
**rs13421194**	TT	250 (80.9)	241 (78.0)	1.122 [0.786-1.603]	0.635	0.526	0.481	0.935	57 (72.2)	181 (85.0)	2.017 [1.157-3.518]	2.474	0.013	0.016	0.048
	TC	54 (17.5)	65 (21.2)						20 (25.3)	30 (14.1)					
	CC	5 (1.6)	3 (1.0)						2 (2.5)	2 (0.9)					

Our study was based on a cohort of relevant sample size and adequately matched taking into consideration known confounding variables. Results here obtained are in agreement with the above-mentioned GWAS, in that rs13421194 was not significantly involved in BU acquisition (OR = 1.193; 95% CI = 0.901-1.579; *p* = 0.214, major allele as reference), although it remains to be known if data on the association with the ulcerative phenotype can also be replicated. Cell death mediators modulate BU pathophysiology and it is now more evident that such process is governed by a complex network of *BCL2L11*-associated SNPs. Because of the low cost and speed of DNA genotyping, the new knowledge here presented may be regarded in the stratification of the risk of ulceration among BU patients. This in turn enables more informed decisions on optimal individual follow-up intervals and on the timing to escalate treatment strategies, potentially modifying the course of the disease.

## Data Availability

The data that support the findings of this study are available from the corresponding author, A.G.F., upon reasonable request. The authors would like to thank all the participants for their openness and patience to participate in the study.

## References

[1] Omansen TF, Erbowor-Becksen A, Yotsu R, et al. Global Epidemiology of Buruli ulcer, 2010-2017, and analysis of 2014 WHO Programmatic Targets. Emerg Infect Dis. 2019;25:2183–2190. 31742506 10.3201/eid2512.190427 PMC6874257

[2] Bieri R, Scherr N, Ruf M-T, et al. The macrolide toxin mycolactone promotes bim-dependent apoptosis in Buruli ulcer through inhibition of mTOR. ACS Chem Biol. 2017;12:1297–1307. 28294596 10.1021/acschembio.7b00053

[3] Capela C, Sopoh GE, Houezo JG, et al. Clinical epidemiology of buruli ulcer from Benin (2005-2013): effect of time-delay to diagnosis on clinical forms and severe phenotypes. PLoS Negl Trop Dis. 2015;9:e0004005. 26355838 10.1371/journal.pntd.0004005 PMC4565642

[4] International HapMap 3 Consortium, Altshuler DM, Gibbs RA et al. Integrating common and rare genetic variation in diverse human populations. Nature 2010; 467: 52–58. 20811451 10.1038/nature09298 PMC3173859

[5] Barrett JC, Fry B, Maller J, et al. Haploview: analysis and visualization of LD and haplotype maps. Bioinforma Oxf Engl. 2005;21:263–265. 10.1093/bioinformatics/bth457 15297300

[6] Johnson JL, Abecasis GR. GAS power Calculator: web-based power calculator for genetic association studies. bioRxiv. 2017. doi:10.1101/164343.

[7] Capela C, Dossou AD, Silva-Gomes R, et al. Genetic variation in autophagy-related genes influences the risk and phenotype of buruli ulcer. PLoS Negl Trop Dis. 2016;10:e0004671. 27128681 10.1371/journal.pntd.0004671 PMC4851401

[8] Purcell S, Neale B, Todd-Brown K, et al. PLINK: a tool set for whole-genome association and population-based linkage analyses. Am J Hum Genet. 2007;81:559–575. 17701901 10.1086/519795 PMC1950838

[9] Yates AD, Achuthan P, Akanni W, et al. Ensembl 2020. Nucleic Acids Res. 2020;48:D682–D688. 31691826 10.1093/nar/gkz966 PMC7145704

[10] Farré D, Roset R, Huerta M, et al. Identification of patterns in biological sequences at the ALGGEN server: PROMO and MALGEN. Nucleic Acids Res. 2003;31:3651–3653. 12824386 10.1093/nar/gkg605 PMC169011

[11] Cooper DN. Functional intronic polymorphisms: Buried treasure awaiting discovery within our genes. Hum Genomics. 2010;4:284–288. 20650817 10.1186/1479-7364-4-5-284 PMC3500160

[12] Yang R, Yang E, Shen L, et al. IL-12+IL-18 Cosignaling in human macrophages and lung epithelial cells activates cathelicidin and autophagy, inhibiting intracellular mycobacterial growth. J Immunol Baltim Md 1950. 2018;200:2405–2417. 10.4049/jimmunol.1701073 PMC5860987 29453279

[13] Russell L, Garrett-Sinha LA. Transcription factor Ets-1 in cytokine and chemokine gene regulation. Cytokine. 2010;51:217–226. 20378371 10.1016/j.cyto.2010.03.006

[14] Kotzin JJ, Spencer SP, McCright SJ, et al. The long non-coding RNA Morrbid regulates Bim and short-lived myeloid cell lifespan. Nature. 2016;537:239–243. 27525555 10.1038/nature19346 PMC5161578

[15] Manry J, Vincent QB, Johnson C, et al. Genome-wide association study of Buruli ulcer in rural Benin highlights role of two LncRNAs and the autophagy pathway. Commun Biol. 2020;3:177. 32313116 10.1038/s42003-020-0920-6 PMC7171125

